# Optimal fibre length and maximum isometric force are the most influential parameters when modelling muscular adaptations to unloading using Hill-type muscle models

**DOI:** 10.3389/fphys.2024.1347089

**Published:** 2024-04-17

**Authors:** James Cowburn, Gil Serrancolí, Steffi Colyer, Dario Cazzola

**Affiliations:** ^1^ Department for Health, University of Bath, Bath, United Kingdom; ^2^ Centre for the Analysis of Motion, Entertainment Research and Applications, University of Bath, Bath, United Kingdom; ^3^ Department of Mechanical Engineering, Universitat Politècnica de Catalunya, Barcelona, Spain

**Keywords:** bed rest, muscle disuse, optimal control problem, direct collocation, Monte Carlo

## Abstract

**Introduction:** Spaceflight is associated with severe muscular adaptations with substantial inter-individual variability. A Hill-type muscle model is a common method to replicate muscle physiology in musculoskeletal simulations, but little is known about how the underlying parameters should be adjusted to model adaptations to unloading. The aim of this study was to determine how Hill-type muscle model parameters should be adjusted to model disuse muscular adaptations.

**Methods:** Isokinetic dynamometer data were taken from a bed rest campaign and used to perform tracking simulations at two knee extension angular velocities (30°·s^−1^ and 180°·s^−1^). The activation and contraction dynamics were solved using an optimal control approach and direct collocation method. A Monte Carlo sampling technique was used to perturb muscle model parameters within physiological boundaries to create a range of theoretical and feasible parameters to model muscle adaptations.

**Results:** Optimal fibre length could not be shortened by more than 67% and 61% for the knee flexors and non-knee muscles, respectively.

**Discussion:** The Hill-type muscle model successfully replicated muscular adaptations due to unloading, and recreated salient features of muscle behaviour associated with spaceflight, such as altered force-length behaviour. Future researchers should carefully adjust the optimal fibre lengths of their muscle-models when trying to model adaptations to unloading, particularly muscles that primarily operate on the ascending and descending limbs of the force-length relationship.

## 1 Introduction

Spaceflight remains an exciting and key objective for international space agencies. In 2018 the International Space Exploration Coordinate Group, a forum for the advancement of global space exploration strategies, published the Global Exploration Road map that outlined long-term aims for space exploration (International Space Exploration Coordinate Group, 2018). Within the road map, the targets of human missions to the Lunar and Mars surfaces in the next 2 decades were described, but importantly crew health and performance was highlighted as a critical target for technological advancement to ensure future missions are successful. The emergence of commercial companies, such as Blue Origin, SpaceX and Virgin Galactic, further adds to the resurgent interest in spaceflight, but also highlights the importance in understanding the influence of spaceflight disuse on the human body. Attenuation of musculoskeletal (MSK) adaptations to spaceflight, including skeletal muscle mass and strength (Winnard et al., 2019), remains a priority to maintain astronaut and passenger health and performance during and following spaceflight. This is further emphasised by NASA’s human research roadmap (National Aeronautical and Space Administration, 2023) and ESA’s SciSpace white papers (European Space Agency, 2021) which highlight the ongoing research priorities to mitigate risks associated with MSK fitness on performance of operational tasks and long-term health. It is clear from these works that both agencies value accurate monitoring of MSK load for personalised and adaptive programming to target emerging adaptations to optimise in-flight and post-flight exercise prescription.

In this context, computational MSK modelling approaches have been identified as a key next step in understanding how exercise in hypogravity can mitigate against and rehabilitate these adaptations (Lang et al., 2017). Our current understanding of MSK loading in hypogravity, both environmental (i.e., spaceflight) and emulated via ground-based facilities (e.g., body weight support systems), has been driven by inverse dynamic calculations of net joint moments and forces (Apte et al., 2018). Relying on net quantities to draw conclusions on voluntary muscle contractions and joint forces is problematic due to the inconsistency between the levels of scale being used to describe the human body. Specifically, net joint moments and forces provide the cumulative influence of all passive and active force generating structures and therefore do not have sufficient architectural or functional scale to determine the contributions of independent elements. Due to challenges in collecting relevant *in vivo* data, computational muscle-tendon unit (MTU) models are necessary to overcome these limitations by representing muscle-activation-contraction dynamics to estimate MTU and joint forces. This information is key to profile the load experienced by the MSK system in hypogravity, and to better align exercise prescription with an individual’s MSK adaptations. This is particularly relevant for post-spaceflight rehabilitation when the functional capacity of an astronaut is most compromised due to the reintroduction of gravitational forces (Berg et al., 1997; English et al., 2020).

Muscle-tendon unit modelling requires representations of neuromechanical, contractile, and passive properties of muscle contraction as mathematical expressions. Simulation of human movement is made possible by coupling these mathematical expressions of force generation with the equations of motion governing the rigid-body system. The complexity of this endeavour is highlighted in the requirement of capturing muscle-tendon physiology across multiple architectural and functional scales to estimate the cumulative effect of the entire muscle. Hill-type models are widely used in muscle-driven simulations due to their flexibility in being fine-tuned depending on the application (Caillet et al., 2022), relative computational efficiency (van den Bogert et al., 1998; Winters, 1990; van Soest et al., 2019), and ability to capture salient features of muscle contraction across multiple scales (Schmitt et al., 2019). However, the phenomenological nature of Hill-type models mean that parameters cannot be attributed to specific muscle structures as in Huxley-type or, to a greater extent, continuum models (Dao and Tho, 2018; Schmitt et al., 2019). In contexts where the aim is to understand the outcome of muscle contraction dynamics at the organ-level on the human body (e.g., load profiling or motor control), particularly in complex multi-body MSK models, the trade-off of complexity for computational efficiency is preferable, and may not sacrifice accuracy of muscle force estimations in certain circumstances (van Soest et al., 2019). Considering estimating MSK load when exercising in hypogravity has been driven by inverse dynamics analyses, estimating muscle forces would be an important advancement in this field to improve exercise prescription. Using the human research roadmap (National Aeronautical and Space Administration, 2023) as an example, the risk of performance declining both during short-term (<30 days) and long-term missions (>30 days), and with exposure to different environments (e.g., Lunar and Mars surfaces), still requires optimisation through improved understanding of MSK load during exercise. Estimating muscle and joint forces would better mitigate against declining operational performance and long-term health due to improve management of MSK fitness.

An important aspect of Hill-type MTU models is the parameter values chosen to describe the contraction dynamics. The Hill-type muscle model has been continuously refined since its original formulation, but is typically described by a contractile element in parallel and in series with force-strain elements that describe the passive structures (Hill, 1938; Winters, 1990; Caillet et al., 2022). The contractile elements contain mathematical expressions for the force-length and force-velocity relationships, together with neuromechanical representations, describing the dependency on activation, length, and velocity on force generation (Zajac, 1989; Caillet et al., 2022). These relationships are normalised by five main muscle-tendon unit (MTU) parameters - maximum isometric force, optimal fibre length, pennation angle at optimal fibre length, tendon slack length, and maximum shortening velocity - to estimate force given the state of the MTU model. Although it is not often considered alongside the five main muscle parameters, the gradient of the tendon force-length relation is important for capturing the influence of stiffness on tendon length, and, subsequently, on muscle length and velocity (Narici and De Boer, 2011). Previous research has shown that the values used for the MTU model parameters can have a substantial influence on the simulated muscle behaviour (Ackland et al., 2012) and estimated muscle forces and joint contact forces (De Groote et al., 2010; Ackland et al., 2012; Valente et al., 2014; Serrancoli et al., 2020). This has important implications for modelling an astronaut post-spaceflight due to muscular adaptations associated with spaceflight. A systematic review of bed rest studies highlighted that, without exercise intervention, muscle atrophy (volume and cross-section area) and weakening can present within as little as 14 days (Winnard et al., 2019). These adaptations are not uniform between muscle groups, with the anti-gravity extensor muscle groups of the legs typically experiencing greater adaptations than flexor groups (Gopalakrishnan et al., 2010; Kramer et al., 2017) and the arm muscles (Rittweger et al., 2005; Gopalakrishnan et al., 2010). Although muscular size and strength are closely linked, the degree of weakening tends to exceed the degree of muscle atrophy following unloading (Alkner and Tesch, 2004). This highlights that additional factors, such as architectural adaptations (i.e., fibre length and pennation angle) and fibre type composition, contribute to the decline in muscular function following unloading. Indeed, shorter and less pennate muscle fibres have been reported alongside reduced muscular strength following spaceflight (Koryak, 2019) and bed rest (Reeves et al., 2002; De Boer et al., 2008). Further evidence of the complexity inherent to muscle adaptations, are i) a preferential shift from type I to type II fibres observed following disuse (Trappe et al., 2004), ii) the inter-individual variation reported in the literature (Fernandez-Gonzalo et al., 2021; Scott et al., 2021), which also complicates the adjustment of muscle model parameters. For example, muscle fibre CSA and peak isometric fibre force ranged between 49%–106% and 30%–90%, respectively, across eight astronauts from a single study (Fitts et al., 2010). Additionally, *in vitro* analyses of single-fibre shortening velocities have been reported to decrease by 44% (Fitts et al., 2010) or to increase two-fold (Yamashita-Goto et al., 2001) following long-term disuse. The appropriateness of the Hill-muscle model for modelling adaptations to unloading is contingent on the ability to measure muscle parameters *in vivo* to personalise the model or identify clear strategies for adjusting parameters depending on the profile of adaptations being modelled.

Modelling approaches are a useful tool that could be used to investigate how the muscles adapt during unloading. Therefore, specific Hill-type muscle model parameters must be adjusted to ensure realism, and validly model different adaptations. The aim of this paper is to evaluate the appropriateness of the Hill-type muscle model in the replication of muscle adaptations due to unloading, and to identify the parameters that are the most important to feasibly model muscular adaptations. A stochastic sampling approach was adopted to randomly sample Hill-type muscle model parameter perturbations to estimate a distribution of feasible combinations for modelling muscular adaptations to unloading.

## 2 Materials and methods

### 2.1 Reference data

The following case study was derived from data presented in the literature. Knee net joint moments recorded at 30° knee flexion during a 30°·s^−1^ and a 180°·s^−1^ angular velocity dynamometry task, pre and post 90-days bed rest, were used as ground truth data (Alkner et al., 2016). These data were chosen as the participants were similar to the participant used in this study (see simulation framework below) These data were combined with normative knee net joint moment profiles of young, healthy adults for the same isokinetic task for flexion angles between 90° and 0° flexion (Knapik et al., 1983). Knapik *et al.* Normative net joint moments reported at 10° intervals were expressed as a fraction of the value at 30° knee flexion. The ground truth net joint moments were multiplied by these fractions and interpolated to construct complete knee extensor moment profiles before and after 90 days bed rest at both angular velocities. These data were used as reference knee net joint moments within the simulation framework.

### 2.2 Simulation framework

Optimal control problems (OCP) were formulated to compute muscle activations and forces. The goal of the OCP was to minimise a cost function (Eq. (1)), subject to MTU activation and contraction dynamics. To investigate the influence of MTU parameters, a Monte Carlo sampling technique was used to randomly perturb MTU parameters prior to input into the OCP. The aim of this approach was to replicate unloading induced adaptations with the objective to identify a hypothetical sets of MTU parameters that represented feasible solutions to the problem.

During isokinetic dynamometry testing, the joint kinematics (positions and velocities) are constrained by the sitting posture and the dynamometer arm. Consequently, we prescribed this information to the model. Similarly, this allowed for the reference net joint moments described above to be used to relate the MTU forces to the kinematics. A generic full-body MSK model was modified before utilisation within the framework (Lai et al., 2017). The model was scaled in OpenSim to an adult male, who had similar anthropometrics to the pre-flight reference data (model participant: 28 years, 79.9 kg, 1.82 m; reference data: 32 ± 4 years, 72 ± 5 kg, 1.73 ± 0.03 m). The torso, both arms, and left leg segments were removed to leave a right-leg only model (assumed to be the tested leg) leaving seven segments (pelvis, femur, patella, tibia-fibula, talus, calcaneus and toes) and 13 degrees of freedom (6 pelvis, 3 hip, and 1 knee, ankle, subtalar and MTP). The remaining knee rotations and translations, and sagittal plane patella kinematics (orientation, and anteroposterior and vertical translations), were parameterised via polynomials as a function of knee angle. It should be noted that the translations were limited to a very narrow range of motion (anteroposterior: 2–5 cm, vertical: ±2 cm). The model was positioned to replicate a sitting posture. Right knee angles were varied from 90° to 0° flexion at 30°·s^−1^ and 180°·s^−1^. Right hip flexion was set to 90°, with the remaining joint coordinates set to anatomical neutral. During initial testing, large parallel elastic component (PEC) forces were observed for the knee flexors as knee flexion angle approached 0°. Consequently, the pelvis was positioned with 30° backwards tilt, and hip flexion adjusted to 60° flexion to maintain a femur position parallel with the ground. This was to allow the musculature to work at more realistic normalised fibre-lengths during the simulations. This also corresponds to a high hip flexion condition in other knee extension dynamometry studies (60°–70°, De Groote et al., 2010). The model was actuated by 40 Hill-type MTUs with the equations governing active (force-length and force-velocity) and passive (parallel and series) force generation according to De Groote et al. (2016, Figure 1). The lengths, velocities and moment arms of the MTUs were described by polynomials as a function of joint angle and joint angular velocity (Van den Bogert et al., 2013; Falisse et al., 2019a). Polynomial coefficients were determined by placing the scaled model in random positions within and exceeding the expected range of motion. Maximum isometric forces were scaled according physiological cross-sectional area (Lieber and Fridén, 2000). Muscle volumes were estimated for the participant’s height and mass (Handsfield et al., 2014) with a specific tension of 60 N·cm^2^, as done previously (Rajagopal et al., 2016). Maximum shortening velocities were assumed to be ten times the optimal fibre lengths, and the tendon force-length curves had a gradient of 35 at 4% strain, as done in a similar simulation framework (Falisse et al., 2019b). Raasch’s activation model (Raasch et al., 1997) was used to model excitation-activation dynamics of the MTUs with modifications by De Groote et al. (2009). Normalised tendon force, $\tilde{F_{t}}$ , and activation, *a*, were used to describe the state of the MTUs, with their first time derivatives introduced as control variables ( ${\overset{\cdot }{\tilde{F}}}_{t}$ and $\overset{\cdot }{a}$ , respectively). A reserve actuator (*τ*_*res*_) was added to the knee flexion-extension coordinate, bounded between ±25 and scaled between ±1. This reserve actuator represents an idealised torque actuator added to augment the muscle forces, allowing the simulation to run when the muscles were unable to produce the required net joint moment.

**FIGURE 1:**
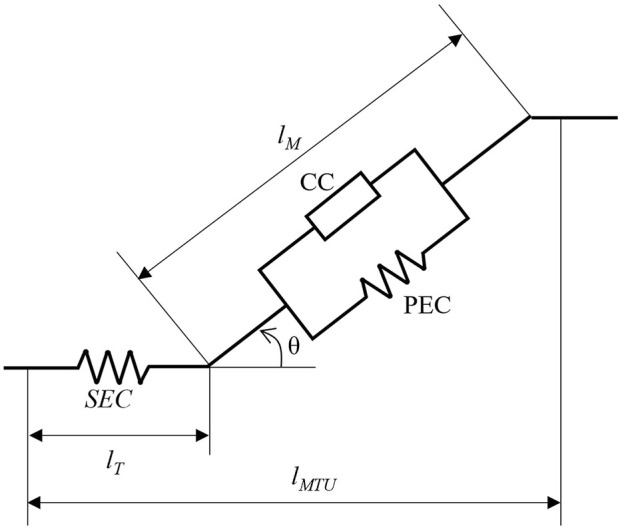
Visual representation of the Hill-type muscle model. The muscle-tendon unit is comprised of three force-generating elements. The series elastic component (SEC), modelling the force-length relationship of the tendon, lies in series with muscle. The muscle activation-length-velocity interaction and the passive force-length interaction are accounted for by contractile component (CC) and the parallel elastic component (PEC), respectively. The muscle is orientated via the pennation angle, *θ*, and determines the contribution of tendon length, *l*_*T*_, and muscle fibre length, *l*_*M*_, to the overall muscle-tendon unit length, *l*_*MTU*_. Schematic modified from De Groote et al. (2016).

A cost function, J, was constructed to solve the muscle redundancy problem through minimising the sum of squared muscle activations (J_*Effort*_), reserve actuator (J_*Reserves*_), and the MTU control variables (J_*Control*_). The sum of muscle activations squared has been used extensively as a surrogate for muscular effort (e.g., Kistemaker et al., 2006; Bayer et al., 2017; Wochner et al., 2020). The activations were multiplied by their muscle volumes, expressed as a percentage of total volume $\left(P_{V_{j}}\right)$ to penalise the use of larger muscles. Despite primarily been used as a surrogate for efficient muscular effort, the sum of muscle activations squared has been successfully used in sprinting simulations (Haralabidis et al., 2021) and was deemed appropriate to use for this maximal knee extension task. Minimisation of control terms was included to improve optimal solutions by penalising large, non-physiological changes in the state variables. The cost function was formulated to minimise the *τ*_*res*_ so that simulations could be identified where reserve actuators did not provide assistance to the muscle forces above a given threshold. The reserve and control variable terms were scaled before inclusion in the cost function.

$$J=\;J_{\mathrm{Effort}}+J_{\mathrm{Reserves}}+J_{\mathrm{Controls}}$$ (1)

$$J_{\mathrm{Effort}}=\overset{40}{\underset{j=1}{\sum }}\int_{t0}^{tf}{\left(P_{V_{j}}\cdot a_{j}\right)}^{2}\;dt$$ (2)

$$J_{\mathrm{Reserves}}=\overset{6}{\underset{i=1}{\sum }}\int_{t0}^{tf}{\left(\tau_{\mathrm{res}}\right)}^{2}\;dt$$ (3)

$$\begin{array}{l} J_{\mathrm{Control}}=\;\overset{40}{\underset{j=1}{\sum }}\int_{t0}^{tf}{\left({\overset{\cdot }{\tilde{F}}}_{t_{j}}\right)}^{2}\;dt+\;\overset{40}{\underset{j=1}{\sum }}\int_{t0}^{tf}{\left(\overset{\cdot }{a_{j}}\right)}^{2}\;dt \end{array}$$ (4)

A direct collocation method was used to transcribe the OCP into NLP using *Legendre-Gauss-Radau* quadrature. Design variables consisted of the state (x = [*a*, ${\tilde{F}}_{t}$ ]) and control (u = [ $\dot{a}$ , $\dot{{\tilde{F}}_{t}}$ , *τ*_*res*_]) variables. The design variable trajectories were discretised into 50 equally spaced mesh intervals (Ackermann and van den Bogert, 2010). State trajectories were further parameterised with third-order polynomials with four points per interval. The framework was implemented in MATLAB (version R2017b, MathWorks Inc, United States of America) and CasADi (Andersson et al., 2019), and was solved using IPOPT (Wachter and Biegler, 2006). A modified OpenSim and Simbody release were utilised to allow for algorithmic differentiation of function derivatives (Falisse et al., 2019a). Path constraints were then imposed at each interval to ensure consistency between the muscle moments and inverse dynamics moments, and the tendon and muscle forces (i.e., Hill-equilibrium). Two inequality constraints were imposed on muscle activations based on activation (*t*_*a*_ = 0.015 s) and deactivation (*t*_*d*_ = 0.06 s) time constants (De Groote et al., 2009). Design variables were scaled between ±1 to improve the numerical conditioning of the NLPs (Betts, 2010). State variable dynamics were enforced at each collocation point, to ensure continuity of the state trajectories. State trajectories at the end of each interval were constrained to ensure continuity.

### 2.3 Monte Carlo framework

A Monte Carlo sampling technique was used to explore MTU adaptations to unloading. For a single MTU, five parameters were identified as inputs for the analysis: maximum isometric force (*F* ^MAX^), optimal fibre length (OFL), penntion angle at OFL (*θ* ^0^), maximum shortening velocity (*V* ^MAX^), and tendon compliance (*k*_*t*_). Tendon slack length was not included for two reasons: i) there is a lack of evidence available describing how tendon length adapts to unloading, and ii) a mechanism for adaptation to a change in loading (e.g., a change in tissue architecture) was not identified to justify its inclusion. In contrast, the tendon has been shown to become more compliant (or less stiff) during unloading (Kubo et al., 2004), which will influence muscle contractions as tendon elongation directly impacts fibre length and velocity (Narici and Maganaris, 2007). Therefore, it was deemed necessary to include tendon compliance to fully understand how Hill-models can replicate adaptations to unloading.

A Monte Carlo simulation was formulated to select parameter perturbations within literature informed boundaries. These boundaries were defined based on spaceflight and ground-based unloading studies to reflect the inter-individual variability that is reported following unloading (Scott et al., 2021), and where necessary, derived based on assumptions about muscle physiology (Lieber and Fridén, 2000). The perturbation boundaries are shown in Table 1.

**TABLE 1:** Boundaries used to perturb the Hill-type muscle model parameters within the Monte Carlo analysis. Values are expressed as a percentage change relative to baseline (i.e., unperturbed = 100%).

Parameter	Perturbation boundaries (%)
Maximum Isometric Force	40–100
Optimal Fibre Length (OFL)	60–100
Pennation Angle at OFL	75–100
Maximum Shortening Velocity	50–200
Tendon Compliance	40–100

**FIGURE 2:**
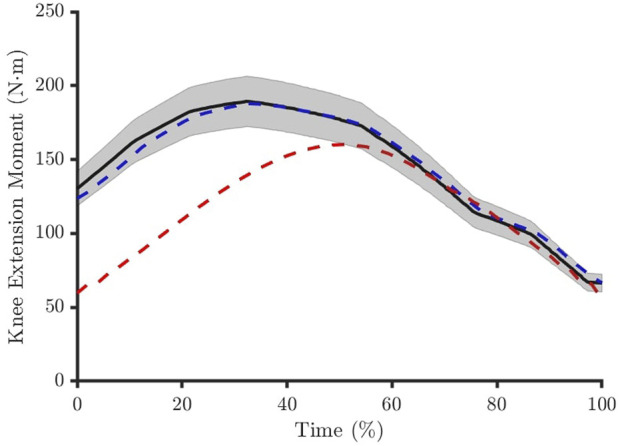
The muscle forces had to produce a net knee joint moment within 9% of the reference experimental data (solid black line and shaded area). The solution was considered feasible if this was achieved across the entire movement period (blue dashed line) and infeasible if this was violated at one or more time points (red dashed line).

Each simulation would then represent the same individual returning to Earth after a hypothetical exposure to microgravity (∼0 g), with all feasible simulations representing a hypothetical population of muscle model parameter combinations that are physiologically meaningful. This would allow for the identification of parameters most important for modelling muscle adaptations and their relative variability in unloading scenarios. Simulations were deemed feasible when it converged to an optimal solution and when reserve actuators did not contribute more than 9% of the reference net joint moment data for the post-unloading condition (Figure 2). The 9% threshold was based on the test-retest reliability of a commercially available dynamometer (Li et al., 1996). All other solutions were defined as infeasible.

To simplify the Monte Carlo simulation, we grouped the 40 MTUs defined in the generic model as flexors, extensors, and non-knee muscles according to their moment arms about the knee joint coordinate. This reduced the number of parameters to perturb from 200 to 15, with five parameters for each muscle groups rather than five for each of the 40 MTUs. Therefore, each muscle parameter within a muscle group was perturbed to the same degree. A set of uniformly distributed values between 0 and 1 were randomly generated in MATLAB. These values were used to derive a percentage perturbation for each input parameter. The baseline values for the MTU parameters were taken from the scaled MSK model used to derive the reference knee net joint moment data in OpenSim. Tendon stiffness and maximum shortening velocity values were set to 35 at 4% strain and 10 times OFL, respectively (Falisse et al., 2019b). A uniform distribution was used to allow for equal probability of perturbations to be sampled across the defined perturbation boundaries. Two Monte Carlo simulations were performed: one for the 30°·s^−1^ condition and one for the 180°·s^−1^ condition. Convergence of each Monte Carlo was defined as when the mean and coefficient of variation of all parameters from the final 10% of simulations was within 2% of the overall mean and coefficient of variation (Ackland et al., 2012; Valente et al., 2014).

### 2.4 Data analysis

All feasible and infeasible solutions were used in further analyses for different angular velocity conditions. The parameter perturbations represented the main outcome measure, and, due to the uniform distribution of the Monte Carlo sampling, data were expressed as median ± inter-quartile range (IQR). Medians, IQRs, and kernel densities were explored between feasible and infeasible solutions to identify patterns in the muscle model parameters. A step-wise logistic regression model was created for each Monte Carlo condition. Parameter perturbations were centred about zero (i.e., unperturbed), and expressed as a percentage to allow regression coefficients to describe odds ratio changes per 1% perturbation of a given parameter. A logit function was used to link the covariates (i.e., the muscle model parameter perturbations and interaction terms) to the log-odds of a simulation being successful, and a maximum likelihood estimation was used to fit the regression model. Terms were added to the model where the adjusted McFadden’s pseudo R^2^ was increased by 2.5%. This threshold was chosen as the best balance between maintaining model simplicity (i.e., fewest necessary predictor variables), and accuracy in classifying parameters as feasible and infeasible. Regression coefficients were transformed into odds ratios, and confidence intervals for the odds ratios were calculated. Confidence intervals that did not cross 1 (i.e., equal odds between feasible and infeasible solutions) were used to identify significant regression terms (*p* < 0.01).

## 3 Results

**FIGURE 3:**
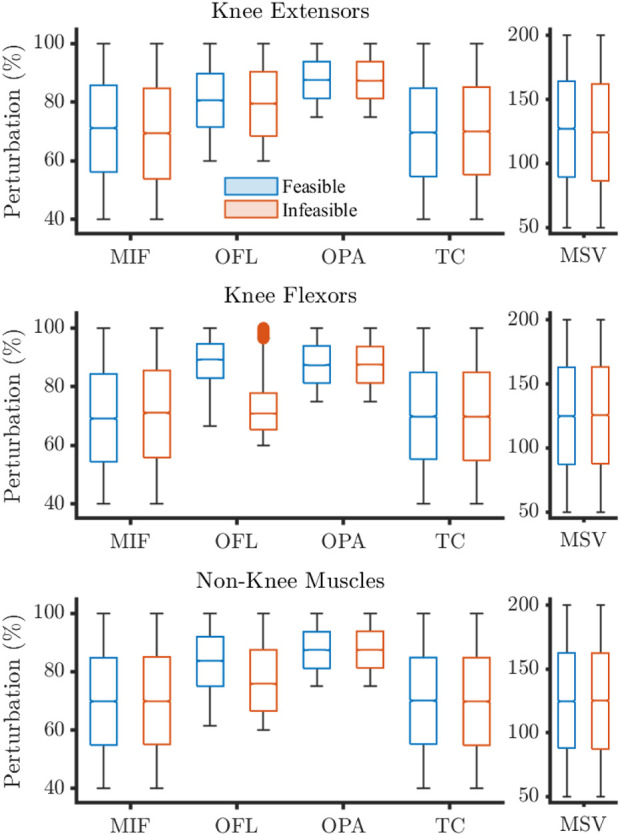
Sampling distributions of parameter perturbations for feasible and infeasible solutions for the 30°·s^−1^ condition. Values expressed as percentages where 100% describes baseline. Filled circles represent outliers. MIF, maximum isometric force; OFL, optimal fibre length; OPA, Pennation angle at OFL; TC, tendon compliance; MSV, maximum shortening velocity.

**FIGURE 4:**
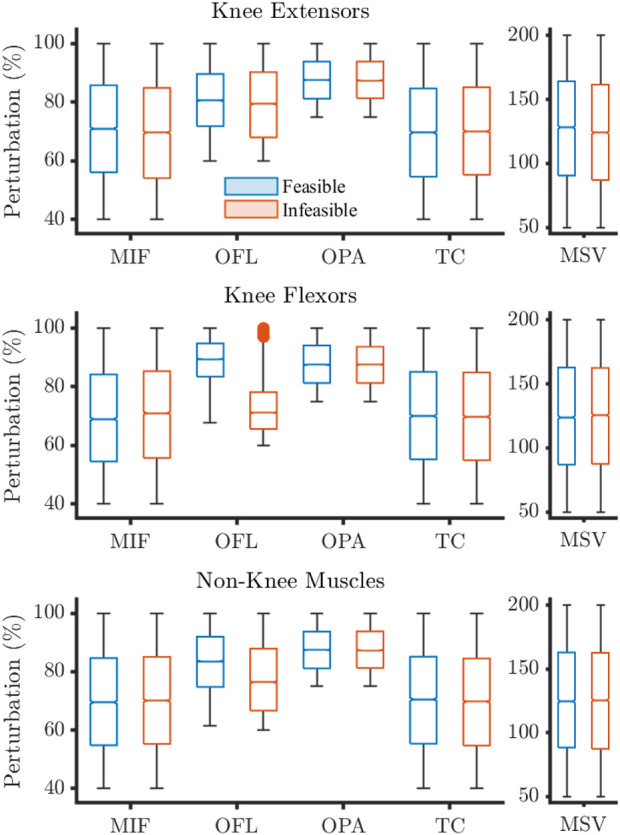
Sampling distributions of parameter perturbations for feasible and infeasible solutions for the 180°·s^−1^ condition. Values expressed as percentages where 100% describes baseline. Filled circles represent outliers. MIF, maximum isometric force; OFL, optimal fibre length; OPA, Pennation angle at OFL; TC, tendon compliance; MSV, maximum shortening velocity.

The distribution of perturbations highlighted differences between the feasible and infeasible solutions for knee flexion and non-knee muscle OFL, and knee extension MIF (Figures 3, 4).

**FIGURE 5:**
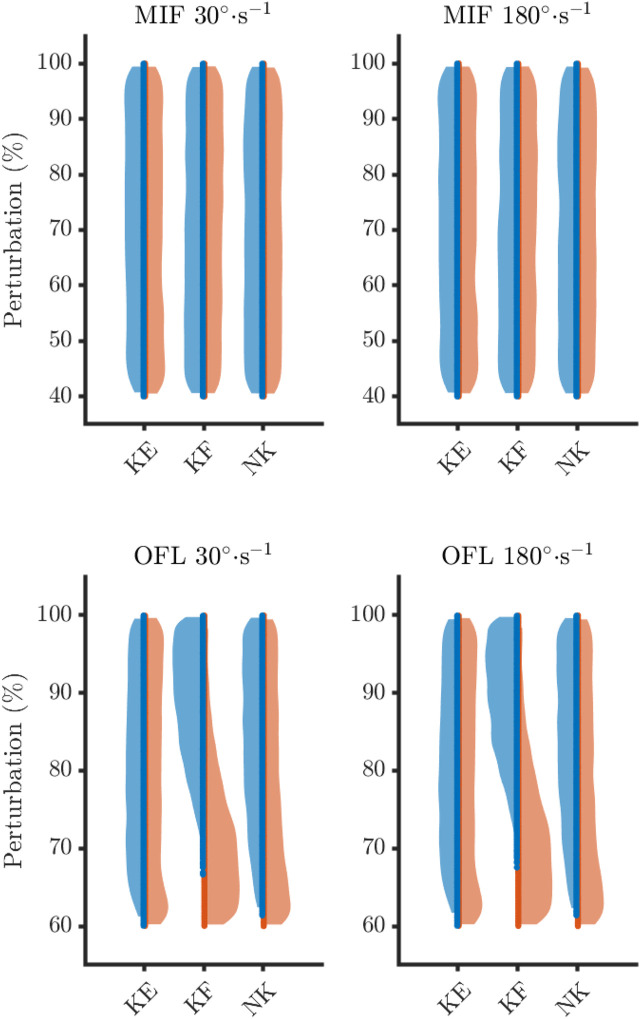
Sampling density of maximum isometric forces (MIF) and optimal fibre length (OFL) for the feasible (blue) and infeasible (orange) solutions. KE, knee extensors; KF, knee flexors; NK, non-knee muscles.

Feasible solutions were more densely populated nearer to baseline for these three parameters (Figure 5), which is reflected in the descriptive statistics.

On average, feasible solutions tended to occur when OFL were not shortened to much for both the knee flexors (30°·s^−1^: feasible = 89.2 ± 5.8%, infeasible = 70.8 ± 6.2%; 180°·s^−1^: feasible = 89.4 ± 5.7%, infeasible = 71.1 ± 6.3) and the non-knee muscles (30°·s^−1^: feasible = 83.7 ± 8.5%, infeasible = 75.8 ± 10.5%; 180°·s^−1^: feasible = 83.6 ± 8.6%, infeasible = 76.4 ± 10.6%). In fact, a maximum level of perturbation was observed for these parameters, as all simulations were categorised as infeasible when the OFL were shortened beyond 67% and 61% of baseline for the knee flexors and non-knee muscles, respectively (Table 2).

**TABLE 2:** The distribution of parameter perturbations expressed as a percentage change relative to baseline (i.e. 100%) for feasible solutions.

		30°·s^−1^					180°·s^−1^				
			Median	IQR	95% CI	Min	Max	Median	IQR	95% CI	Min	Max
Knee Extensors	MIF	71.2	14.8	70.6–71.7	40.0	100.0	71.0	15.0	70.3–71.6	40.0	100.0
	OFL	80.6	9.1	80.2–80.8	60.0	100.0	80.6	8.9	80.1–81.0	60.0	100.0
	OPA	87.6	6.3	87.3–87.8	75.0	100.0	87.6	6.3	87.3–87.8	75.0	100.0
	MSV	127.3	37.4	126.1–128.3	50.0	200.0	128.2	36.8	126.6–129.6	50.0	200.0
	TC	69.6	15.1	69.1–70.1	40.0	100.0	69.6	15.1	68.9–70.2	40.0	100.0
Knee Flexors	MIF	69.1	15.0	68.6–69.6	40.0	100.0	68.9	14.9	68.3–69.5	40.0	100.0
	OFL	89.2	5.8	89.0–89.3	66.7	100.0	89.4	5.7	89.2–89.6	67.4	100.0
	OPA	87.4	6.3	87.2–87.7	75.0	100.0	87.5	6.4	87.2–87.8	75.0	100.0
	MSV	124.7	37.9	123.2–125.9	50.0	200.0	123.8	37.9	122.2–125.5	50.0	200.0
	TC	69.9	14.8	69.4–70.4	40.0	100.0	70.0	14.9	69.4–70.6	40.0	100.0
Non-Knee Muscles	MIF	69.8	15.0	69.4–70.3	40.0	100.0	69.5	14.9	68.8–70.1	40.0	100.0
	OFL	83.7	8.5	83.5–84.0	61.4	100.0	83.6	8.6	83.3–83.9	61.4	100.0
	OPA	87.4	6.3	87.2–87.6	75.0	100.0	87.4	6.3	87.1–87.8	75.0	100.0
	MSV	124.6	37.2	123.3–125.8	50.0	200.0	124.6	37.3	123.1–126.1	50.0	200.0
	TC	70.1	14.8	69.6–70.6	40.0	100.0	70.5	14.9	69.8–71.1	40.0	100.0

MIF, maximal isometric force; OFL, optimal fibre length; OPA, pennation angle at OFL, TC, tendon compliance; MSV, maximum shortening velocity; CI, confidence interval, Min and Max, minimum and maximum perturbations that led to feasible solutions.

Feasible solutions were obtainable across the entire spectrum of perturbation values for the remaining muscle parameters. The density of feasible and infeasible solutions were qualitatively similar and evenly spread across the perturbation ranges for the remaining solutions.

Both logistic regression models were exclusively comprised of OFL parameters (Table 3). Optimal fibre lengths of the knee flexor and non-knee muscles were the strongest predictors of achieving feasible simulations. For every percentage the OFL parameters were increased the odds ratios increased by 22% (30°·s^−1^) and 22% (180°·s^−1^) for the knee flexors OFL, and 10% (30°·s^−1^) and 9% (180°·s^−1^) for the non-knee OFL. The intercept values from both models demonstrated that when the other predictor variables were zero (i.e., the baseline parameters were used) the odds ratio of a feasible solution was substantial. The adjusted R^2^ values for the final models were 0.59 and 0.58 for the 30°·s^−1^ and 180°·s^−1^ conditions, respectively.

**TABLE 3:** Included parameters in the logistic regression models with coefficients expressed as odds ratios and odds ratio 99% confidence intervals.

	Log odds	Odds ratio	Confident interval
30°·s^−1^			
Intercept	6.16	475.46	(375.68–601.74)
KF OFL	0.22	1.25	(1.24–1.26)
NK OFL	0.10	1.10	(1.10–1.11)
180°·s^−1^			
Intercept	5.82	336.11	(251.77–448.70)
KF OFL	0.22	1.25	(1.24–1.26)
NK OFL	0.09	1.09	(1.09–1.10)

OFL, Optimal fibre length KF, knee flexors; NK, non-knee muscles.

**FIGURE 6:**
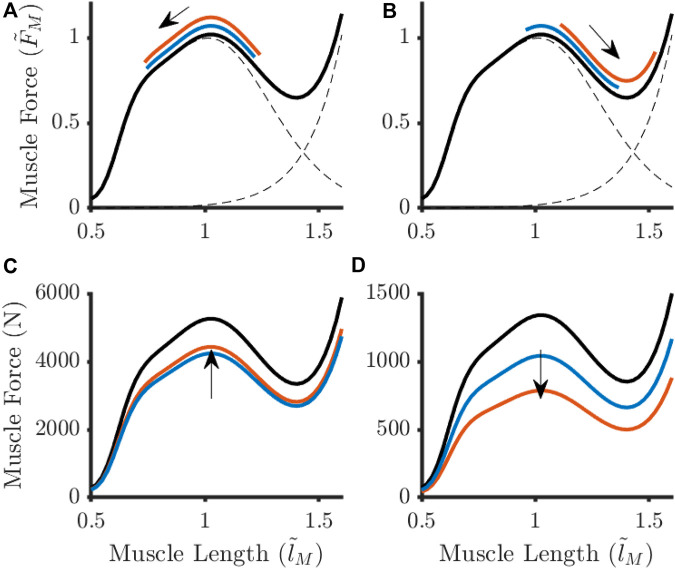
Adaptations to the baseline force-length relationship (black lines) for an example knee extensor **(A, C)** Vastus Lateralis) and knee flexor **(B, D)** Biceps Femoris Long Head) in response to optimal fibre length shortening at 30°·s^−1^. Different parameter adjustment strategies arose when large perturbations (orange) to optimal fibre length were observed in comparison to when this parameter remained unchanged (blue). The arrows represent the direction fibre length changes during knee extension **(A, B)** and the shift in maximum isometric force **(C, D)**. Muscle force were normalised to Maximum isometric force ( $\tilde{F_{M}}$ in **(A, B)**.

When OFL was shortened, the knee flexor muscles were forced to work within the descending limb of the force-length relationship (Figure 6). In this situation, it was observed that MIF needed to decrease proportionally for feasible solutions to be obtained. This pattern was not observed within the knee extensor muscles.

## 4 Discussion

The aim of this study was to understand how Hill-type muscle model parameters can be adjusted to reflect unloading-induced muscular adaptations. To do so, kinematics and kinetics data of knee flexion-extension performed on a dynamometer, post-spaceflight, were retrieved from the literature and used for the analysis. A Monte Carlo sampling technique was used to randomly perturb muscle model parameters and understand their importance in modelling muscular adaptations to disuse. The findings showed that the Hill-type muscle model can be used to replicate physiological muscular adaptations providing that adjustments to the knee flexor and non-knee muscle optimal fibre lengths, and the knee extensor maximum isometric forces, are appropriate. This provides guidance for future researchers and clinicians on how to calibrate MTU parameters given the emerging adaptations of an astronaut. This is an important next step for space agencies, as highlighted by the Human Research Roadmap (National Aeronautical and Space Administration, 2023) and SciSpace White papers (European Space Agency, 2021), as more accurate estimation of MSK loading via muscle and joint forces will better mitigate against declining operational performance and long-term health due to improve management of MSK fitness.

The simulations that were optimal solutions represented feasible parameter sets for reproducing the post-disuse knee joint torque. The likelihood of attaining such optimal solution was most influenced by the knee flexor and non-knee muscle optimal fibre lengths. Specifically, shortening the optimal fibre lengths reduced the odd ratios for both the 30°·s^−1^ and 180°·s^−1^ conditions. This supports previous studies that have highlighted that it is important to calibrate optimal fibre length to obtain valid simulation outcomes (Scovil and Ronsky, 2006; Redl et al., 2007; De Groote et al., 2010; Ackland et al., 2012; Serrancoli et al., 2020). It was unexpected that maximum isometric force was not included within the step-wise regression models given the maximum effort nature of the dynamometer task. There is debate within the literature as to whether simulation results are (Scovil and Ronsky, 2006; De Groote et al., 2010) or are not sensitive (Redl et al., 2007; Ackland et al., 2012) to maximum isometric force. Those studies that found maximum isometric force was an important MTU parameter were investigating movements that required high force output. For example, Scovil and Ronsky (2006) found that simulations of running were sensitive to maximum isometric force but walking simulations were not. While in the most similar study to this, where the sensitivity of net joint moments to MTU parameters was assessed during maximal isometric and isokinetic knee extension dynamometry tasks, it was suggested that sensitivity to optimal fibre length and maximum isometric force was substantial for some muscles (De Groote et al., 2010). On face value, it is possible that the maximum isometric forces were not perturbed enough to prevent the muscles being able to balance the net joint moments. However, given the boundaries were informed by the disuse literature and the lower boundary represented 40% of the unperturbed values, this is difficult to envisage. Alternatively, this finding may be an artifact of the muscle sharing term aiming to minimise the muscle activations. We observed that the knee flexors, particularly the hamstring muscles, were on average not activated above 20% and spent the majority of the movement below 10% of maximum activation. Minimal active force generation from the knee flexors will have made it easier for the knee extensors to balance the net knee joint moment, reducing the need for large maximum isometric forces. The minimising of muscle activations means the simulations assumed minimal co-activation. However, antagonist co-activation is a known phenomenon, particularly for joint stability (Neptune et al., 1999), and can be considerable in the hamstrings during isokinetic knee extension dynamometry (e.g., Osternig et al., 1986). One approach that can better represent co-contraction is EMG-informed modelling (e.g., Pizzolato et al., 2015). Future research should consider alternative muscle sharing terms within the cost function or utilise EMG-informed modelling (e.g., Pizzolato et al., 2015) to better represent co-activation.

Without other similar modelling studies for comparison, the logical question is then whether the adjustments presented in this study represent ‘appropriate’ adaptations to the MTU parameters to model unloading adaptations. Maximum isometric force is commonly assumed to be proportional to physiological cross-sectional area, and regularly scaled in MSK models based on this assumption (e.g., Reeves et al., 2005). The maximum perturbation allowed for MIF within the Monte Carlo was determined using this method, and is therefore reflective of literature data that would be used to scale MIF in a MSK model. It is worth noting the specific tension value used within this study (60 N·cm^−2^) to estimate the “pre-flight” maximum isometric forces. While this value was chosen to be consistent with that used to develop the MSK model (Rajagopal et al., 2016), it is greater than values typically reported for lower-limb muscles (e.g., 15–30 N·cm^−2^ Erskine et al., 2009; 2010; Maganaris et al., 2001). Overestimating the specific tension will have led to overestimating the maximum strength of the muscles and subsequent muscles forces. However, this overestimation was applied across all muscles, and it is not expected to have influenced the perturbation behaviour for the MIF. To discuss optimal fibre length, it is important to consider what is being modelled by this parameter. For a single sarcomere, the optimal length represents the point at which the maximum number of cross-bridges can be formed due to overlap of actin and myosin filaments (van den Bogert et al., 1998; Gordon et al., 1966). However, sarcomeres are not necessarily uniformily spaced within a fiber (Huxley and Peachey, 1961; Llewellyn et al., 2008; Moo et al., 2016), meaning it is plausible that architectural adaptations to unloading will alter the fiber length at which the maximum number of cross-bridges can be formed. Indeed, there is consistent evidence of reduced muscle CSA (Winnard et al., 2019) and fascicle length changes (Reeves et al., 2002; De Boer et al., 2008; Rittweger et al., 2018; Koryak, 2019) after unloading, which suggest a loss of sarcomeres both in parallel and series formation. This can manifest in reduced functional capacity, with astronauts presenting with reduced sit-and-reach performance upon return-to-Earth relative to pre-flight (Laughlin et al., 2015), likely due to a more flexed resting posture in microgravity (i.e., ∼0 g) (Simons, 1964; Han Kim et al., 2019). It has been hypothesised that reduced serial sarcomeres would result in a right-shift on the force-length curve at equivalent MTU lengths because the sarcomere would be lengthened to achieve the same fascicle length (Narici and Maganaris, 2007). The results of this study showed that shortening the OFL, indicative of serial sarcomere loss, recreated the right-shift on the force-length curve. Further, feasible solutions were observed when OFLs were unchanged or shortened within a muscle-group, albeit to different levels of tolerance. The ability to recreate features of muscle contraction dynamics and align with literature measured outcomes (i.e., reduced flexibility) provide evidence that shortening OFL is a likely physiological adaptation to unloading. Utilising post-spaceflight assessment processes to determine fascicle length (e.g., using ultrasound) would allow for identifying whether adjustment of OFL is necessary to appropriately use the Hill-type model to recreate muscular adaptations to unloading.

Having established that optimal fibre length is a realistic adaptation, the next question is whether it is physiologically plausible that optimal fibre-length can shorten by as much as 21% in the knee flexors. No feasible solutions were observed when perturbing the OFL beyond 79% and 76% of baseline values for the knee flexors and non-knee muscles, respectively, suggesting there is a physiological limit that cannot be exceeded for the hypothetical data used in this study. Assuming that structural adaptations alone govern the change in OFL (i.e., fascicle length is directly proportional to OFL), the most perturbed value reported in this study falls within fascicle length changes reported following bed rest and spaceflight (−26% and 0%, Rittweger et al., 2018; De Boer et al., 2008; Koryak, 2019; Reeves et al., 2002). Although it is important to note that only one study reported fascicle length changes in excess of −10% (−5 to −26%, Koryak, 2019). These results suggest that there should be inter-individual variation in OFL and that OFL shortening will not be excessive in the majority of individuals. The study results support these suggestions as feasible solutions were achieved between −21% and 0% for the knee flexor muscles and −24% and 0% for the non-knee muscles, but with a higher density of feasible solutions at lower levels of perturbation for both muscle groups. However, whether salient features of an astronauts post-spaceflight movement patterns can be captured is difficult to ascertain because there is a sparsity of biomechanical data available and few previous modelling studies to compare OFL adjustments used to model adaptations to the muscular system. Drawing from another clinical population, the more flexed posture of the hip and knee joints in microgravity is analogous, albeit less extreme, to the crouched gait position reported in Cerebral Palsy children (Wren et al., 2005). Crouch gait occurs, in part, due to muscle contractures (i.e., muscle shortening) of the iliopsoas and hamstring musculature (Delp et al., 1996; Schutte et al., 1997). Shortening OFL, in isolation or alongside lengthening tendon slack length, has been a common approach for simulating contractures in this context. Predictive simulations have shown that crouch gait emerges when the OFL of the correct hip and knee flexor muscles is shortened between −40% and −50% (Mehrabi et al., 2019; Falisse et al., 2020). Given crouch gait represented an extreme context for which OFL adjustments may be necessary to model adaptations of the underlying pathology, it seems reasonable that modelling adaptations to unloading did not reach this magnitude of shortening. The challenge for future research is to determine whether the characteristics of an astronauts movement, such as during gait, are captured with the MTU parameter adjustments outlined in this study.

Similar to maximum isometric force, maximum shortening velocity, pennation angle at OFL nor tendon compliance were found to be important parameters for modelling adaptations to disuse. Given the six-fold change in angular velocity between conditions, it is logical to hypothesise that MSV would be more influential at 180°·s^−1^ where MTU length changes may occur more rapidly. Increasing MSV allows muscles to generate greater forces at similar contractile component (CC) shortening velocities. Compliance of the series elastic component (SEC) can facilitate CC-SEC dynamics by permitting the CC to work at comparable shortening velocities even when MTU shortening velocities may be different (Miller et al., 2012). The Monte Carlo simulations could only perturb tendon stiffness by making the tendon more compliant, and it is possible that the more compliant tendon allowed for the CC to work within similar force-velocity regions. However, since optimal solutions were found across the entire range of the MSV and tendon compliance (TC) perturbations, it appears that the baseline TC value was sufficient to allow the CC to work in an appropriate region of the force-velocity relationship. Pennation angle was not found to be influential in this study, likely because it was only allowed to decrease when perturbed. Decreasing the pennation angle increases the proportion of the muscle forces (active and passive) projected along the tendon, meaning perturbing this parameter was reducing the muscle force requirements to achieve the same joint moment contribution. However, this does align with previous observations of Hill-type formulations, which generally consider pennation angle to have relatively little influence on muscle forces when ≤20° (Zajac, 1989). Of the 40 muscles in the model, 35 had a baseline optimal pennation angles of ≤20° , which would only become smaller once perturbed during the Monte Carlo simulation. These results suggest that to model adaptations to unloading, it is more important to appropriately calibrate MIF and OFL to model the architectural adaptations to the muscle. However, it may still be necessary to adjust these parameters to investigate MTU behaviour changes following spaceflight. The complexity of the muscular system allows other parameters within the same muscle model (Miller et al., 2012), or forces from other muscles (van der Krogt et al., 2012), to compensate for perturbations to a given parameter. It is likely that the adaptations to maximum shortening velocity, pennation angle at OFL and tendon compliance are specific to the individual, and future researchers should consider their unique research questions when deciding which parameters to adjust alongside MIF and OFL.

There are limitations of the approach taken in this study that should be recognised when interpreting the results. In the absence of experimental data, the reference net joint moments derived for the knee extension task do not have corresponding EMG data to validate muscle activations. Nonetheless, salient features of muscle activity, such as post-disuse presenting with decreased activations (Kramer et al., 2018) and a tendency for greater activity from vastus lateralis relative to the rectus femoris (Salzman et al., 1993), were captured by the simulations. The shape and magnitude of the activations cannot be further verified, and thus it cannot be ruled out that muscle activation amplitude did not compensate for parameter alterations. Additionally, neural adaptations were omitted from the study as the main focus was on modelling the muscular adaptations of the MSK system to disuse. Alterations to the neural dynamics of the muscle have been shown through reduced amplitude of EMG (Berg et al., 1997; Kramer et al., 2018), greater torque generation via supramaximal stimulation than voluntary contraction (Koryak, 2014), and altered firing frequency and fibre conduction velocity (Mulder et al., 2009). However, muscle force estimations are relatively insensitive to the time of activation and deactivation (Scovil and Ronsky, 2006), and it is expected there would have been minimal influence on the conclusions of this study. Furthermore, the nature of adaptations within muscles groups was assumed to be uniform to reduce the complexity of the Monte Carlo simulation. However, heterogeneity within muscle groups in how the muscles adapt has been observed in the literature (e.g., LeBlanc et al., 2000). It is unclear whether muscles within a muscle group were more sensitive to the parameter perturbations than others, which prevents conclusions being drawn as to whether parameters within the same group should be adjusted differently. It should also be recognised that the underlying MSK model was primarily developed based on male participants, validated against a single male and single female participant, scaled to a male participant, and the reference isokinetic knee moment data were derived from a sample of male participants. The results of this study need to be replicated for female derived data to have full confidence in the applicability of the result to women. Finally, tendon slack length was not included in the Monte Carlo analysis because we could not find any evidence within the literature to show a change with unloading, nor a physiological mechanism that might explain how this parameter may adapt to a change in (un)loading conditions. However, it is recognised that tendon slack length is regularly cited as being an important parameter for determining muscle forces in computational approaches (e.g., Hicks et al., 2015; Serrancoli et al., 2020). It is recommended that for modelling adaptations to unloading using the Hill-type muscle model, tendon slack length should not be changed once the pre-spaceflight value has been determined (e.g., after calibration using computational approaches).

It has been shown that the Hill-type muscle model is capable of modelling muscular adaptations to unloading. It is important to carefully adjust optimal fibre length, particularly for muscles working within the ascending and descending limbs of the force-length relationship, to obtain feasible simulation results. Shortening the optimal fibre length aligns with observations that returning from spaceflight is associated with a more flexed posture and loss of sarcomere in series, but extreme shortening can influence the ability to obtain realistic simulation results. Future work should consider investigating more movements (e.g., gait or jumping movements) and alternative muscle sharing terms within the objective function to further understand how the Hill-type-muscle model can be used to represent adaptations to disuse.

## Data Availability

The original contributions presented in the study are included in the article/supplementary material, further inquiries can be directed to the corresponding author.
